# Fecal *Cloacibacillus porcorum* Improves Non-Invasive Diagnosis of Colorectal Adenoma in the Hong Kong Population

**DOI:** 10.3390/ijms27104457

**Published:** 2026-05-15

**Authors:** Yao Zeng, Effie Yin Tung Lau, Silin Ye, Jiawei Lu, Rui Zhang, Ruoyu Hu, Jessie Qiaoyi Liang

**Affiliations:** 1 Department of Medicine and Therapeutics, Li Ka Shing Institute of Health Sciences, The Chinese University of Hong Kong, Hong Kong, China; yaozeng@link.cuhk.edu.hk (Y.Z.); effielau@cuhk.edu.hk (E.Y.T.L.); silinye@link.cuhk.edu.hk (S.Y.); jiawei.lu@link.cuhk.edu.hk (J.L.); ruizhang2025@link.cuhk.edu.hk (R.Z.); ruoyuhu@link.cuhk.edu.hk (R.H.); 2 Shenzhen Research Institute, The Chinese University of Hong Kong, Shenzhen 518063, China

**Keywords:** bacterial biomarkers, marker panel, *Cloacibacillus porcorum*, non-invasive diagnosis, colorectal cancer and adenoma

## Abstract

We previously developed a four-marker panel for the diagnosis of colorectal cancer (CRC) and adenoma. This study aimed to identify novel bacterial markers to improve adenoma detection using metagenomics and qPCR. Candidate markers were identified from metagenomic data (*n* = 492) using ANCOM-BC2 and Spearman’s rank correlation analysis and were subsequently validated in an independent cohort (*n* = 426). Diagnostic performance was assessed both individually and in combination with our previously identified markers and FIT. Metagenomic analysis identified 21 candidate markers that increased along the normal–adenoma–carcinoma axis. Two top candidates, *Cloacibacillus porcorum* (Cp) and *Intestinimonas butyriciproducens*, were validated via qPCR and showed significant correlations with metagenomic abundances (both *p* < 0.0001). ROC analysis demonstrated that Cp levels significantly distinguished CRC and adenoma from controls, whereas *I*. *butyriciproducens* distinguished only CRC. The prevalence of Cp was significantly higher in adenoma and CRC than in controls (all *p* < 0.05). Multivariate analysis confirmed that Cp was independently associated with CRC and adenoma diagnoses. Adding Cp to the four-marker panel improved diagnostic sensitivity from 44.8% to 58.7% for adenoma and from 85.7% to 88.6% for CRC (specificity = 85%). When further combined with FIT, Cp improved sensitivity from 47.6% to 64.3% for adenoma and from 95.2% to 96.2% for CRC (specificity = 84.6%). *C*. *porcorum* is a novel bacterial marker that may aid in the non-invasive diagnosis of colorectal adenoma.

## 1. Introduction

Colorectal cancer (CRC) is one of the most common malignancies worldwide [1]. Most CRCs begin as small polyps. Some polyps, particularly adenomas, gradually develop into cancer. Early cancer detection can facilitate successful treatment, whereas early adenoma detection can prevent CRC and reduce its incidence. However, although currently available non-invasive CRC screening tests perform well in detecting CRC, their sensitivity for adenoma detection remains limited. CRC incidence is higher in more developed regions than in less developed regions, and this increased incidence is believed to be associated with dietary differences [2,3]. Recent evidence has shown that an altered gut microbiome is associated with colorectal tumorigenesis. Abnormalities in gut microbiota composition have been implicated as potentially important etiological factors in the initiation and progression of CRC [4]. With the widespread application of metagenomic analyses to investigate the intestinal microbiota, an increasing number of bacterial species have been identified as being positively associated with CRC [5,6,7,8]. Recent basic research has established a critical role for the intestinal microbiota [9] and specific bacterial species, such as *Fusobacterium nucleatum* (*Fn*) [10,11,12], in promoting colorectal tumorigenesis. Bacteria such as *Fn* [13], *Clostridium symbiosum* [14], and species within the genera *Parvimonas*, *Porphyromonas*, and *Parabacteroides* [15] have been identified as potential markers for the CRC diagnosis. However, current knowledge regarding biomarkers for colorectal adenoma detection remains limited.

We previously identified and validated bacterial markers for the non-invasive diagnosis of CRC and adenoma using metagenome sequencing and targeted qPCR [5,13]. Specifically, we developed a qPCR test targeting four bacterial markers for CRC and adenoma diagnosis: Fn, the bacterial gene marker m3, *Clostridium hathewayi* (Ch), and *Bacteroides clarus* (Bc) [16]. Three of these markers (Fn, m3, and Ch) are enriched in the stools of patients with CRC or adenoma, whereas Bc is enriched in healthy individuals. Although this four-marker panel demonstrates superior performance compared with currently available non-invasive diagnosis tests for CRC and adenoma, further improvement in adenoma detection sensitivity is still needed. Therefore, this study aimed to identify additional bacterial species based on metagenomic analyses that could enhance adenoma diagnosis and to develop new bacterial marker panels for CRC and adenoma using targeted quantification via qPCR.

## 2. Results

### 2.1. Metagenomic Analysis Identifies Bacterial Species Enriched Along the Normal–Adenoma–Carcinoma Sequence

**Figure 1:**
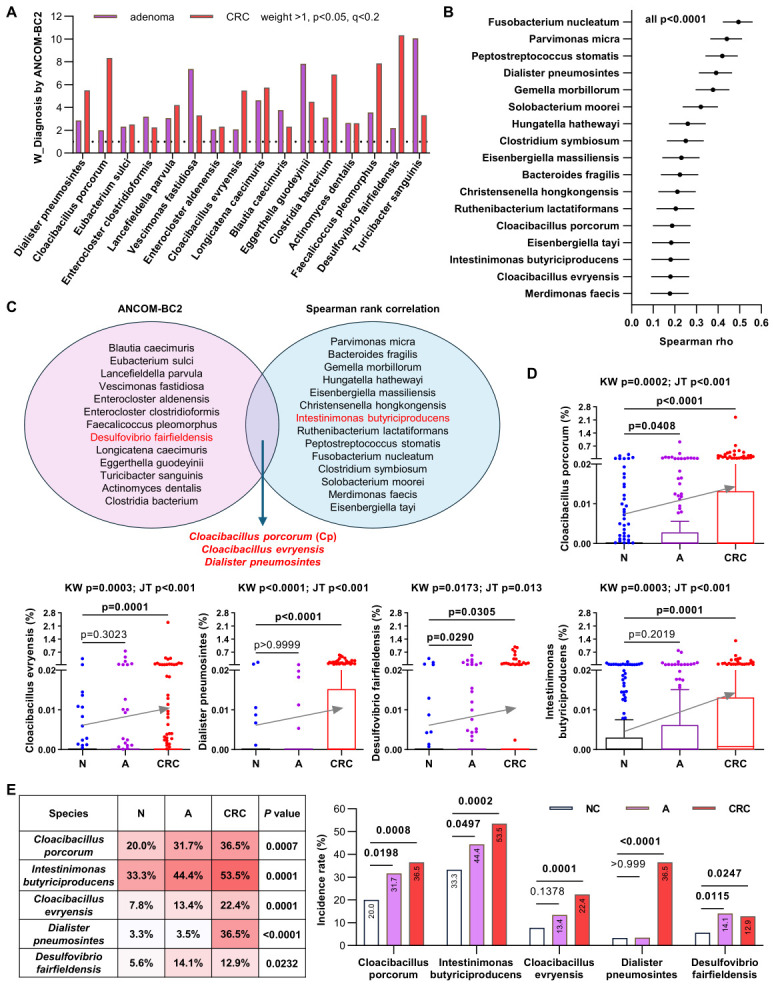
(**A**) Identification of 16 candidate species that are increased in patients with adenoma or CRC compared with control subjects, as determined by ANCOM-BC2. (**B**) Seventeen candidate species exhibited increasing abundance trends from normal to adenoma and CRC, as assessed by Spearman’s rank correlation analysis. (**C**) Three candidate species were identified by both ANCOM-BC2 and Spearman’s rank correlation analyses. (**D**) Comparison of the relative abundances of five selected candidates among the CRC, adenoma and normal control groups. (**E**) Comparison of the incidence rates of five selected candidate species among the CRC, adenoma, and normal control groups, analyzed using Chi-square tests (**left**) and Fisher’s exact tests (**right**). N, normal control; A, adenoma. KW, Kruskal–Wallis test with Dunn’s correction; JT, Jonckheere–Terpstra trend test.

To identify bacteria enriched during normal–adenoma–CRC progression as potential biomarkers for CRC screening, we analyzed metagenomic data from 180 controls, 142 patients with adenoma, and 170 patients with CRC in a Hong Kong Chinese population. ANCOM-BC2 identified 16 species with known classifications that were significantly elevated in both adenoma and CRC compared with normal controls (Figure 1A). Spearman’s rank correlation analysis identified 17 candidate species exhibiting increasing trends across normal–adenoma–carcinoma progression (*p* < 0.0001; Figure 1B). Both ANCOM-BC2 and Spearman’s rank correlation analyses consistently identified three species enriched across disease stages: *Cloacibacillus porcorum* (Cp), *Cloacibacillus evryensis*, and *Dialister pneumosintes* (Figure 1C). Among these, only Cp was significantly increased in both adenoma and CRC compared with normal controls, whereas *C. evryensis* and *D. pneumosintes* were not significantly increased in adenoma compared with controls (Figure 1D). Among the identified candidates, 21 species exhibited increasing trends along the normal–adenoma–carcinoma axis, as determined by the Jonckheere–Terpstra trend test (Table S1), and their relative abundances were further compared between groups (Figure S1). *Desulfovibrio fairfieldensis*, identified by ANCOM-BC2, was significantly increased in both adenoma and CRC compared with normal controls. *Intestinimonas butyriciproducens*, identified by Spearman’s rank correlation analysis, exhibited an increasing trend, although the increase in adenomas compared with controls was not statistically significant (Figure 1D). Among these five candidates, the incidence rates of Cp, *D. fairfieldensis*, and *I. butyriciproducens* were significantly higher in the adenoma and CRC groups than in the control group (Figure 1E). Because the incidence rate of *D. fairfieldensis* was less than 15% in both the adenoma and CRC groups, we focused on evaluating Cp and *I. butyriciproducens* as potential candidates for the non-invasive diagnosis of CRC and adenoma.

### 2.2. Quantification of Bacterial Candidates via qPCR

**Figure 2:**
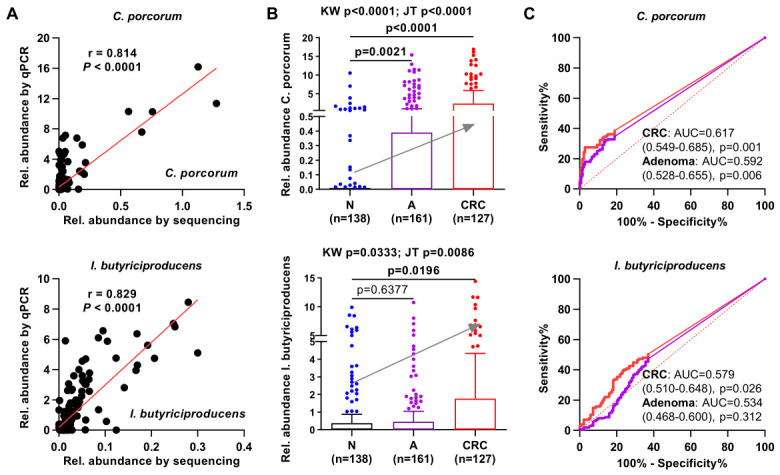
(**A**) Correlations between quantification by metagenome sequencing and qPCR for *C. porcorum* and *I. butyriciproducens*. (**B**) Comparison of *C. porcorum* and *I. butyriciproducens* among the CRC, adenoma, and normal control groups, as assessed via qPCR. (**C**) Evaluation of the diagnostic performance of *C. porcorum* and *I. butyriciproducens* for CRC and adenoma using ROC curve analysis. N, normal control; A, adenoma; AUC, area under the curve. Grey arrows indicate the increasing trend from N to CRC.

Cp and *I. butyriciproducens* have not previously been used as biomarkers for the diagnosis of colorectal neoplasms. Therefore, we established qPCR assays for their targeted quantification. Primers and probes were designed to target the selected species-specific marker genes based on MetaPhlan. The qPCR results exhibited significant positive correlations between the quantification of the two species and their relative abundances obtained from metagenome sequencing (*n* = 280; both *p* < 0.0001; Figure 2A). The qPCR results further demonstrated that the relative levels of both Cp and *I. butyriciproducens* significantly differed among the three groups. However, only Cp was significantly increased in both adenoma and CRC samples compared with controls (Kruskal–Wallis test, *p* < 0.05; Figure 2B). Importantly, the relative level of Cp assessed by qPCR also showed a significant increasing trend from normal to adenoma and CRC (*p* < 0.0001 by the Jonckheere–Terpstra trend test). ROC analysis indicated that Cp could significantly distinguish CRC and adenoma from controls, whereas *I. butyriciproducens* could only distinguish CRC from controls (Figure 2C). These results highlight the potential of Cp as a non-invasive diagnostic marker for CRC and adenoma.

### 2.3. Fecal C. porcorum Shows No Significant Difference Between Advanced and Non-Advanced Adenomas or Across CRC Stages

**Figure 3:**
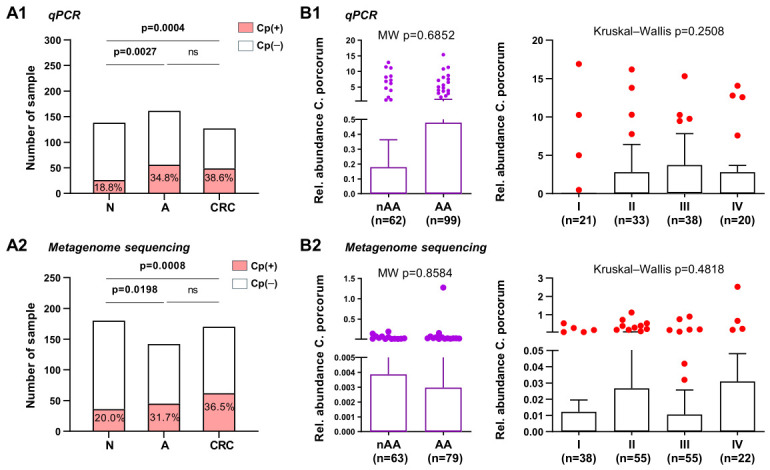
(**A**) Fecal prevalence of *C. porcorum* was significantly higher in the adenoma and CRC groups than in the control group, as assessed via qPCR (**A1**) and metagenome sequencing (**A2**). (**B**) Fecal levels of *C. porcorum* showed no significant differences between non-advanced adenoma (nAA) and advanced adenoma (AA), or among TNM stages, as assessed via qPCR (**B1**) and metagenome sequencing (**B2**). N, normal control; A, adenoma. Samples without TNM stage information were excluded from the staging-based comparisons. ns, not significant.

According to the qPCR results, the prevalence of Cp was significantly higher in patients with adenoma and CRC than in normal controls (Figure 3(A1)), consistent with the metagenome sequencing results (Figure 3(A2)). The relative level of Cp, which was significantly elevated in patients with adenoma or CRC compared with controls, showed no significant difference between non-advanced and advanced adenomas or among different TNM stages, as assessed via qPCR (Figure 3(B1)). These findings are consistent with the metagenome sequencing results (Figure 3(B2)).

### 2.4. Fecal C. porcorum Is an Independent Marker Associated with CRC and Adenoma Diagnosis

**Figure 4:**
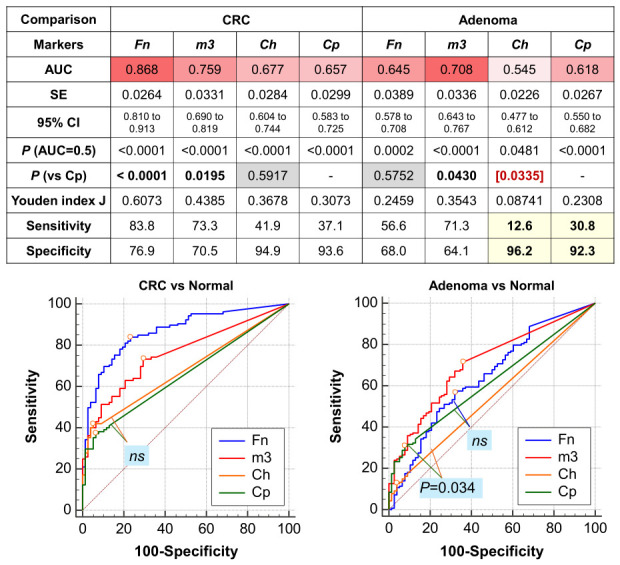
Comparison of the diagnostic performance of individual bacterial markers for CRC and adenoma using ROC curve analysis and pairwise ROC curve comparisons. Fn, *Fusobacterium nucleatum*; Ch, *Clostridium hathewayi*; *m3*, marker m3; Cp, *C. porcorum*; AUC, area under the receiver operating characteristic curve.

We further assessed the diagnostic performance of Cp using qPCR and compared it with the three CRC-enriched bacterial markers (Fn, m3, and Ch) from our previous 4Bac panel [16]. Fecal Cp alone yielded AUCs of 0.657 (95%CI: 0.583 to 0.725; *p* < 0.0001) for CRC and 0.618 (95%CI: 0.550 to 0.682; *p* < 0.0001) for adenoma (Figure 4). At cutoff values maximizing the Youden index, Cp showed sensitivities of 37.1% at a specificity of 93.6% for CRC and 30.8% at a specificity of 92.3% for adenoma. We then compared Cp with the markers Fn, m3, and Ch. For distinguishing CRC from controls, Cp was less effective than Fn and m3 and showed no significant difference compared with Ch. For distinguishing adenoma from controls, m3 performed best, whereas Cp showed no significant difference compared with Fn and was significantly better than Ch (*p* = 0.0335) (Figure 4).

The relative levels of all four markers (Fn, m3, Ch, and Cp) were significantly associated with CRC and adenoma diagnosis, and none were associated with sex, CRC stage, lesion location, or body mass index in univariate analysis (Table S2). While Fn, m3, and Ch significantly increased with age, Cp showed no association with age. Multivariate analysis confirmed that Fn, m3, Ch, and Cp were all independently associated with CRC and adenoma diagnosis, while Fn was also associated with age (Table S3). Further univariate and multivariate analyses of diagnostic factors confirmed that the diagnostic value of Cp is also independent of other markers (Table S4). Logistic regression further demonstrated that Cp is an independent marker for the diagnosis of both adenoma and CRC (Table S5).

### 2.5. Combination of C. porcorum with Other Bacterial Markers Increases Diagnostic Performance for CRC and Adenoma

**Figure 5:**
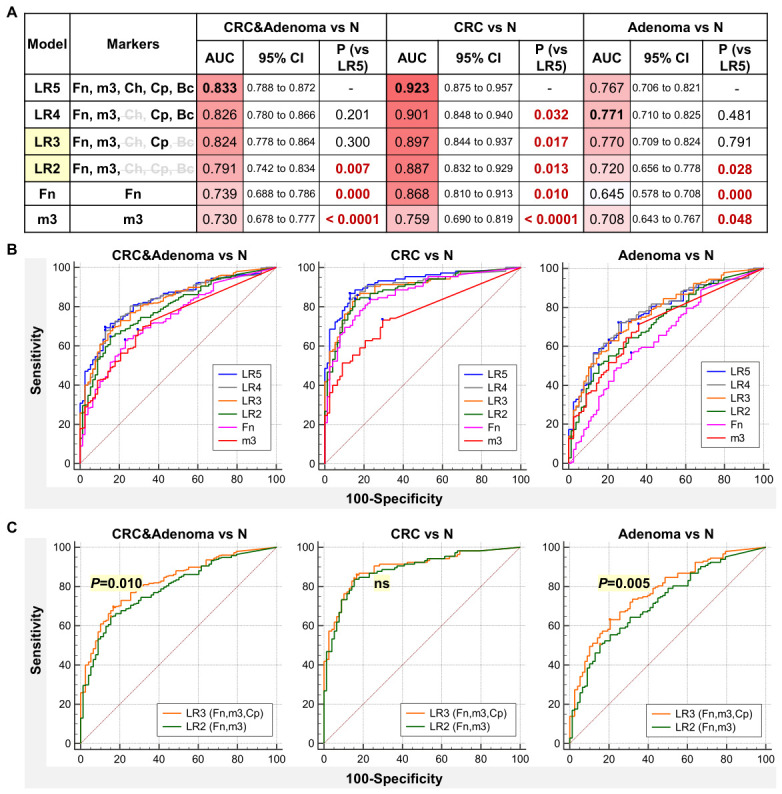
Diagnostic performance of bacterial marker panels for CRC and adenoma using ROC curve analysis. (**A**) Performance parameters of various logistic regression models based on five microbial markers. (**B**) ROC curves comparing the 5-marker model (LR5) with sub-models. (**C**) ROC curves comparing LR2 (Fn, m3) and LR3 (Fn, m3, Cp) to show the incremental diagnostic value of Cp. LR, logistic regression; N, normal control; AUC, area under the receiver operating characteristic curve; Fn, *Fusobacterium nucleatum*; Ch, *Clostridium hathewayi*; *m3*, marker m3; Cp, *C. porcorum*; Bc, *Bacteroides clarus*.

We further assessed combinations of Cp with markers from our previous 4Bac panel (Fn, Ch, m3, and Bc) for CRC and adenoma diagnosis (Table S6). Logistic regression models were constructed to distinguish patients with CRC/adenoma cases from controls, incorporating all five markers or subsets thereof, with individual markers sequentially removed based on their importance in the model. The results showed that the five-marker model (Fn, Ch, Bc, m3, and Cp) performed best for CRC diagnosis, achieving an AUROC of 0.923 (all *p* < 0.05 compared with combinations containing fewer markers, as demonstrated by ROC curve comparisons; Figure 5A,B). For adenoma, the five-marker model showed no significant difference compared with the four- and three-marker panels when *Ch* and/or *Bc* were removed (Figure 5A,B). Comparisons between the two- and three-marker models, involving Fn and m3 with and without Cp, showed no significant difference for CRC diagnosis. However, inclusion of Cp significantly improved diagnostic performance for adenoma (Figure 5A,C).

### 2.6. C. porcorum Significantly Improves the Diagnosis of Adenoma by 4Bac and m3

**Figure 6:**
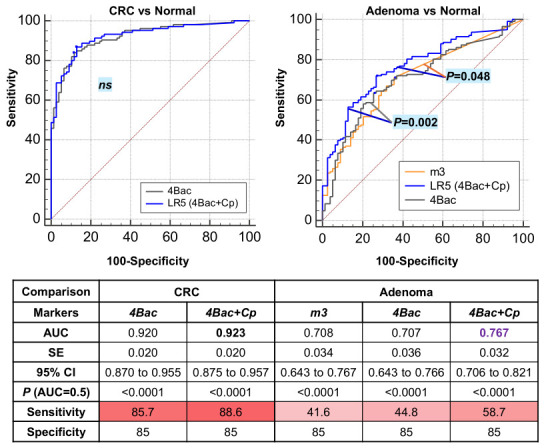
*C. porcorum* significantly improved adenoma diagnosis, as determined by the ‘4Bac’ test and marker m3. 4Bac: *Fusobacterium nucleatum*, *Clostridium hathewayi*, marker m3, and *Bacteroides clarus*; Cp, *C. porcorum*; AUC, area under the receiver operating characteristic curve.

We further compared the diagnostic performance of the 5Bac model incorporating Cp with the 4Bac model and m3, the key marker for adenoma diagnosis. Although the 5Bac model (4Bac + Cp) showed no significant difference compared with 4Bac in CRC diagnosis (*p* > 0.05 by ROC curve comparison), it exhibited slightly higher sensitivity (88.6% vs. 85.7%) at 85% specificity (Figure 6). For adenoma diagnosis, the addition of Cp significantly improved the diagnostic performance of 4Bac (*p* = 0.002 by ROC curve comparison). Additionally, the 5Bac model performed significantly better than *m3* alone for adenoma (*p* = 0.048). At 85% specificity, the 5Bac model showed higher sensitivity for adenoma (58.7%) than 4Bac (44.8%) and *m3* (41.6%) (Figure 6).

### 2.7. Combination of FIT and C. porcorum Enhances the Diagnostic Performance of 4Bac for CRC and Advanced Adenoma

**Figure 7:**
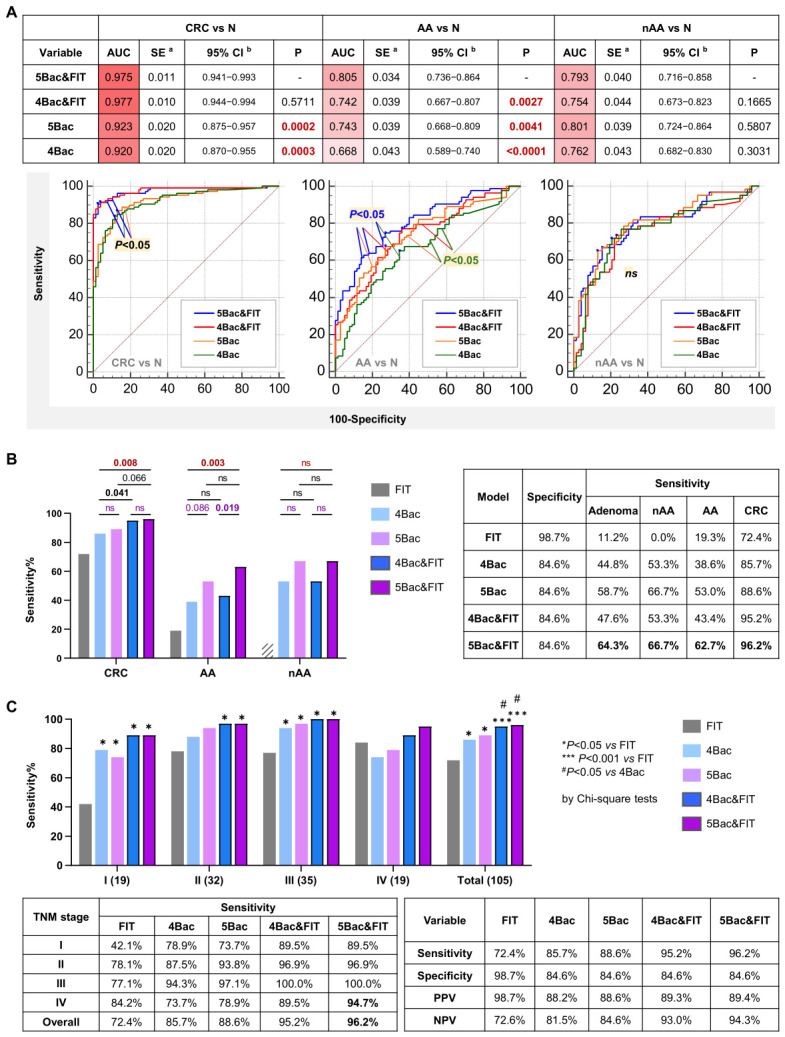
(**A**) Combination with the fecal immunochemical test (FIT) significantly improved diagnostic performance for CRC and advanced adenoma. (**B**) Effects of FIT and *C. porcorum* on CRC and adenoma detection. (**C**) Comparison of the sensitivities of FIT, bacterial markers, and their combinations for CRC detection according to TNM stage subsets. 4Bac: *Fusobacterium nucleatum*, *Clostridium hathewayi*, marker m3, and *Bacteroides clarus*; 5Bac: 4Bac and *C. porcorum*; AA, advanced adenoma; nAA, non-advanced adenoma; N, normal control; AUC, area under a receiver operating characteristic curve. PPV, positive predictive value; NPV, negative predictive value.

We further evaluated the role of Cp combined with FIT in improving the diagnostic performance of the 4Bac model for CRC and adenoma. Logistic regression models were constructed by combining bacterial markers with FIT to distinguish CRC or adenoma cases from normal controls. Performance was assessed separately for CRC, AA, and nAA, as FIT can detect a small proportion of AA but not nAA [16]. When combined with FIT, the 5Bac model (4Bac + Cp) showed no significant difference compared with 4Bac in CRC diagnosis (5Bac + FIT vs. 4Bac + FIT). However, both models performed significantly better than their corresponding models without FIT (both *p* < 0.001 by ROC curve comparisons) (Figure 7A). For advanced adenoma, both FIT and Cp significantly improved the diagnostic performance of 4Bac (both *p* < 0.05), and the 5Bac + FIT model performed significantly better than all other models (all *p* < 0.05) (Figure 7A). For non-advanced adenoma, no significant differences were observed among the models; however, at a given specific specificity (e.g., >80%), models including Cp showed higher sensitivity (Figure 7A).

In our tested cohort, FIT alone detected 72.4% of CRC, 19.3% of AA, and none of the non-advanced adenoma cases at a specificity of 98.7% (Figure 7B). Comparison between 5Bac + FIT and 4Bac showed that the inclusion of Cp and FIT significantly increased detection rates for CRC and AA (both *p* < 0.01). When comparing models with and without FIT, it was evident that FIT improved CRC detection (*p* = 0.041 for 4Bac + FIT vs. 4Bac; *p* = 0.066 for 5Bac + FIT vs. 5Bac). When comparing models with and without Cp, it was evident that Cp improved detection rates for AA and nAA, although the increase was significant only for AA (*p* = 0.019 for 5Bac + FIT vs. 4Bac + FIT) (Figure 7B).

The diagnostic sensitivities of FIT, bacterial markers, and their combinations for CRC were further compared across TNM stage subsets (Figure 7C). The bacterial marker models demonstrated higher sensitivities than FIT for stage I–III cancers but not for late-stage IV disease. The combination of 4Bac or 5Bac with FIT significantly increased sensitivity for stage I–III cancers and also improved detection of stage IV cancers, although not significantly. Cp improved the sensitivity of 4Bac for detecting stage II–IV cancers, although not significantly. These results demonstrate that bacterial marker panels outperform FIT for detecting stage I–III CRC, and their combination further enhances non-invasive CRC diagnosis.

## 3. Discussion

In this study, we identified novel bacterial species markers for the diagnosis of CRC and adenoma using metagenomic analysis and further validated them through targeted quantification using qPCR. Among the identified and validated candidate species, *C. porcorum* emerged as the most promising marker, showing significant increases in both adenoma and CRC samples compared with controls. We compared the diagnostic performance of Cp with that of our previously identified bacterial markers, including m3, Fn, Ch, and Bc. Additionally, we developed new bacterial marker panels, with or without FIT, for the diagnosis of CRC and adenoma. Our findings indicated that the addition of Cp significantly enhanced the diagnostic performance of the previously identified bacterial markers for CRC and adenoma, including both non-advanced and advanced adenomas, whereas combining these markers with FIT further improved the diagnosis of CRC and advanced adenoma.

Targeted detection of bacterial markers identified through shotgun metagenomics represents a promising strategy for clinical application due to its cost-effectiveness and ease of implementation. In this study, qPCR-based quantification of the bacterial markers demonstrated good performance for the diagnosis of CRC and adenoma. In particular, the panel including Fn, m3, and Cp yielded AUCs of 0.897 (95%CI: 0.844 to 0.937) for CRC and 0.770 (95%CI: 0.709 to 0.824) for adenoma. The further addition of Bc and/or Ch increased the AUCs for CRC to above 0.92. Combining these markers with FIT further increased the diagnostic sensitivities for CRC and advanced adenoma; however, detection of non-advanced adenomas was not improved due to the known limitations of FIT in this group. The optimal panel, which included all five bacterial markers and FIT, achieved sensitivities of 64.3% for adenoma and 96.2% for CRC without compromising specificity. This improvement was primarily driven by Cp, which is independent of fecal hemoglobin and other bacterial markers. By detecting non-bleeding or FIT-negative lesions, Cp may help address a critical diagnostic gap in early adenoma screening. The maintenance of high specificity (84.6%) alongside improved detection suggests that this approach may offer a favorable net clinical benefit without imposing an excessive burden on downstream colonoscopy services. However, its meaningful clinical utility needs to be evaluated in future prospective studies to confirm its real-world effectiveness and cost-efficiency by assessing downstream colonoscopy demand and net clinical benefit.

Because the true performance of the markers cannot be accurately determined from case–control samples alone, rigorous external validation in large, independent, multi-ethnic cohorts is essential to ensure robustness and generalizability across diverse screening populations. Given the single-time-point sampling design of this study, longitudinal studies are needed to confirm the temporal stability of Cp. Such studies should also collect detailed dietary and medication histories to control for potential transient confounding factors. Although some normal control individuals showed relatively high levels of Cp, these individuals also tended to have elevated levels of other CRC-associated bacteria included in the 4Bac panel; therefore, adding Cp improved sensitivity without compromising the specificity of the 4Bac panel. In our cohort, multivariate and logistic analyses confirmed that Cp was independently associated with CRC and adenoma and showed no significant association with potential confounding factors, including age, sex, body mass index, CRC stage, or lesion location. Samples in this study were collected either before colonoscopy or one month afterward, when the gut microbiome would be expected to have recovered to baseline [17]. Potential confounding factors, including bowel preparation, dietary patterns, underlying disease conditions, and population heterogeneity, may influence microbiome profiles. Future prospective studies should rigorously control for these variables to confirm the specificity of Cp. The current study focused on individuals aged ≥50 years, as this age group represents the primary target population for routine CRC screening. However, the performance of Cp as a diagnostic marker for CRC and adenomas in individuals younger than 50 years remains unclear, particularly among high-risk groups with early-onset CRC, such as those with hereditary cancer syndromes. Future studies involving younger populations are therefore warranted to further define the diagnostic utility of Cp for early-onset CRC.

Cp, a mucin-degrading bacterium, was first isolated from the intestinal tract of swine [18]. Its role in humans remains largely unknown, and it has primarily been reported in animals. Following fecal microbiota transplantation that improved chronic diarrhea in a cynomolgus monkey, the relative abundance of Cp in the gut microbiota was significantly reduced [19]. As a mucin-degrading bacterium, enrichment of Cp may compromise the integrity of the intestinal mucus barrier, thereby potentially facilitating the translocation of pro-inflammatory factors or pathogens that promote epithelial cell transformation. Notably, Cp has been associated with epizootic rabbit enteropathy and bacteriemia in humans [20,21], supporting its potential role as an opportunistic pathogen. These findings suggest a potential pathogenic role for Cp. However, further in vitro and in vivo functional investigations are warranted to determine whether Cp contributes to colorectal tumorigenesis.

## 4. Materials and Methods

### 4.1. Metagenomics Dataset

We analyzed fecal metagenomic sequencing data from 492 Hong Kong Chinese subjects, including 170 patients with CRC, 142 with adenoma (63 with non-advanced adenoma (nAA) and 79 with advanced adenoma (AA)), and 180 control subjects [22]. Relative species abundances were determined using MetaPhlAn (version 4.0, Segata Lab, University of Trento, Trento, Italy) [23].

### 4.2. Human Fecal Sample Collection

Fecal samples were collected from 426 Hong Kong Chinese subjects (127 with CRC, 161 with adenoma, and 138 normal controls) at the Prince of Wales Hospital, the Chinese University of Hong Kong, between 2009 and 2019. Subjects recruited for fecal sample collection included individuals presenting with symptoms such as a change in bowel habits, rectal bleeding, abdominal pain, or anemia, as well as asymptomatic individuals aged 50 years or above undergoing screening colonoscopy, as described in our previous metagenomic study [5]. Samples were collected either before colonoscopy or one month afterward, when the gut microbiome would be expected to have recovered to baseline [17]. The exclusion criteria were as follows: (1) use of antibiotics within the previous 3 months; (2) adherence to a vegetarian diet; (3) invasive medical intervention within the previous 3 months; (4) a history of cancer or inflammatory disease of the intestine. Subjects were instructed to collect stool samples in standardized containers at home and immediately store them in their home freezer at −20 °C. Frozen samples were then delivered to the hospitals in insulating polystyrene foam containers and immediately stored at −80 °C until further analysis. Patients were diagnosed by colonoscopic examination and histopathological review of biopsy specimens, when applicable. Informed consent was obtained from all subjects. The study was approved by the Joint Chinese University of Hong Kong, New Territories East Cluster Clinical Research Ethics Committee (the Joint CUHK-NTEC CREC, CREC Ref. Nos.: 2021.126 and 2017.369).

### 4.3. DNA Extraction, Design of Primers and Probes, and qPCR

DNA extraction, design of primer and probe sequences, and qPCR amplifications on an ABI QuantStudio sequence detection system were performed as previously described [13]. Primer and probe sequences specifically targeting *C. porcorum* and other markers are listed in Table S7. Primer and probe sequences targeting other bacterial gene markers and the 16s rDNA internal control were adopted from our previous study [13]. Each probe carried a 5′ reporter dye FAM (6-carboxy fluorescein) or VIC (4,7,2′-trichloro-7′-phenyl-6-carboxyfluorescein) and a 3′ quencher dye TAMRA (6-carboxytetramethyl-rhodamine). Primers and hydrolysis probes were synthesized by Invitrogen (Carlsbad, CA, USA). PCR amplification specificity was confirmed by direct Sanger sequencing of PCR products or by sequencing randomly selected TA clones. The relative level of each marker was calculated using the delta Cq method relative to the internal control and expressed as the log value of ‘*10 × 10^6^ + 1’.

### 4.4. Fecal Immunochemical Test (FIT)

Stool samples (*n* = 326) were examined using FIT with the automated quantitative OC-Sensor test (Eiken Chemical, Tokyo, Japan). The quantitative OC-Sensor test was performed as previously described [24], with a positive cutoff value equivalent to 100 ng hemoglobin per milliliter.

### 4.5. Scoring Algorithms and Cutoff Values

The combined score for four bacterial markers (4Bac) using a logistic regression model (4Bac score = I_1_ + β_1_ × *Fn* + β_2_ × *m3* + β_3_ × *Bc* + β_4_ × *Ch*) was determined in a previous study [16]. The combined scores for two to five markers with or without FIT using logistic regression models are listed in Table S8. In the regression models, ‘I’ represents the intercept, ‘β’ represents the regression coefficients, and the markers represent the corresponding Cq values. Cutoff values were determined by receiver operating characteristic (ROC) curve analyses that maximized the Youden index (J = Sensitivity + Specificity − 1) [25].

### 4.6. Statistical Analyses

Values are expressed as mean ± SD or median (interquartile range (IQR)), as appropriate. Differences in bacterial abundances were determined using the Mann–Whitney U test for comparisons between two groups or the Kruskal–Wallis test with Dunn’s correction for comparisons among three groups. The Jonckheere–Terpstra trend test was used to evaluate changes in marker levels during disease progression from control to adenoma to CRC. Simple and multiple regression analyses were performed to estimate associations between marker levels and factors of interest. Incidence rates among different groups and sensitivities of different markers were analyzed using Fisher’s exact test (two groups) or the Chi-square test (three groups). Combinations of multiple biomarkers were evaluated using logistic regression models to generate values estimating disease incidence relative to controls. ROC curves were used to evaluate the diagnostic performance of bacterial markers and models in distinguishing CRC or adenoma from controls. Pairwise comparisons of ROC curves were performed using a nonparametric approach [26]. All analyses were conducted using Graphpad Prism 9.5 (Graphpad Software Inc., San Diego, CA, USA) or MedCalc Statistical Software version 18.5 (MedCalc Software bvba, Ostend, Belgium; http://www.medcalc.org; 2018). *p <* 0.05 was considered statistically significant.

## 5. Conclusions

This study identified *C. porcorum* as a valuable marker for the non-invasive diagnosis of CRC and adenoma using metagenomic analysis. Additionally, we developed a new bacterial marker panel based on targeted quantification via qPCR, which has potential for implementation in CRC screening.

## Data Availability

The data presented in this study have been included in the paper. Other data are available on request from the corresponding author.

## Supplementary Materials

The following supporting information can be downloaded at https://www.mdpi.com/article/10.3390/ijms27104457/s1.

## Abbreviations

Fn	*Fusobacterium nucleatum*
Ch	*Clostridium hathewayi*
Bc	*Bacteroides clarus*
CRC	Colorectal cancer
FIT	Fecal immunochemical test
Cp	*Cloacibacillus porcorum*
*C. porcorum*	*Cloacibacillus porcorum*
*I. butyriciproducens*	*Intestinimonas butyriciproducens*
nAA	Non-advanced adenoma
AA	Advanced adenoma
*D. fairfieldensis*	*Desulfovibrio fairfieldensis*
