# Supplementary material for: Fecal Cloacibacillus porcorum Improves Non-Invasive Diagnosis of Colorectal Adenoma in the Hong Kong Population
Source: Int J Mol Sci. 2026 May 15;27(10):4457. doi: 10.3390/ijms27104457 (PMC13207295; doi:10.3390/ijms27104457)
Supplement: Supplementary file 1 [file ijms-27-04457-s001.zip › ijms-4207175-supplementary tables.pdf]

Table S1. Jonckheere–Terpstra trend test for identified candidates across the normal–adenoma–carcinoma axis.

#	species_name	Std. J-T Statistic	Asymp. Sig. (2-tailed)
1	s__Bacteroides_fragilis	4.883	**<0.0001**
2	s__Christensenella_hongkongensis	4.75	**<0.0001**
3	s__Cloacibacillus_evryensis	3.988	**<0.0001**
4	s__Cloacibacillus_porcorum	4.195	**<0.0001**
5	s__Clostridium_symbiosum	5.607	**<0.0001**
6	s__Desulfovibrio_fairfieldensis	2.491	**0.0127**
7	s__Dialister_pneumosintes	8.664	**<0.0001**
8	s__Eisenbergiella_massiliensis	5.102	**<0.0001**
9	s__Eisenbergiella_tayi	4.062	**<0.0001**
10	s__Enterocloster_aldenensis	3.566	**0.0004**
11	s__Enterocloster_clostridioformis	2.738	**0.0062**
12	s__Faecalicoccus_pleomorphus	3.004	**0.0027**
13	s__Fusobacterium_nucleatum	10.985	**<0.0001**
14	s__Gemella_morbillorum	8.391	**<0.0001**
15	s__Hungatella_hathewayi	5.721	**<0.0001**
16	s__Intestinimonas_butyriciproducens	4.034	**<0.0001**
17	s__Merdimonas_faecis	3.925	**<0.0001**
18	s__Parvimonas_micra	9.864	**<0.0001**
19	s__Peptostreptococcus_stomatis	9.327	**<0.0001**
20	s__Ruthenibacterium_lactatiformans	4.556	**<0.0001**
21	s__Solobacterium_moorei	7.107	**<0.0001**
22	s__Actinomyces_dentalis	0.392	0.695
23	s__Blautia_caecimuris	0.744	0.457
24	s__Clostridia_bacterium	1.759	0.078
25	s__Eggerthella_guodeyinii	0.718	0.472
26	s__Eubacterium_sulci	0.257	0.798
27	s__Lancefieldella_parvula	1.904	0.057
28	s__Longicatena_caecimuris	0.288	0.773
29	s__Turicibacter_sanguinis	1.475	0.140
30	s__Vescimonas_fastidiosa	-0.596	0.551

Table S2. Univariate correlations between fecal marker abundances and clinical characteristics.

Variable*	*Fn*		*m3*		*Ch*		*Cp*	
	**coef**	***P***	**coef**	***P***	**coef**	***P***	**coef**	***P***
**Age**	0.274	<0.001	0.19	<0.001	0.138	0.014	0.052	0.359
**Sex**	-	0.679	-	0.319	-	0.386	-	0.053
**Diagnosis**	0.481	<0.001	0.347	<0.001	0.339	<0.001	0.238	<0.001
**CRC staging**	0.043	0.675	-0.1	0.343	0.098	0.333	0.065	0.522
**CRC location**	-	0.317	-	0.695	-	0.398	-	0.764
**Adenoma location**	-	0.519	-	0.063	-	0.23	-	0.131
**BMI**	0.031	0.729	0.029	0.74	0.116	0.52	-0.03	0.861

**Fn*, *Fusobacterium nucleatum*; *m3*, marker m3; *Ch*, *Clostridium hathewayi*; *Cp*, *Cloacibacillus porcorum*; BMI, body mass index.

Table S3. Multivariate correlations between fecal marker abundances and clinical characteristics.

Variable*	*Fn*		*m3*		*Ch*		*Cp*	
	**coef**	***P***	**coef**	***P***	**coef**	***P***	**coef**	***P***
**Age**	0.083	0.017	0.048	0.132	0.013	0.398	-0.010	0.634
**Diagnosis**	2.174	<0.001	1.299	<0.001	0.511	<0.001	0.645	<0.001

**Fn*, *Fusobacterium nucleatum*; *m3*, marker m3; *Ch*, *Clostridium hathewayi*; *Cp*, *Cloacibacillus porcorum*.

Table S4. Univariate and multivariate analyses of factors associated with diagnosis.

**Variables***	**Univariate**			**Multivariate**		
	**Coefficient**	**95% CI**	**P**	**Coefficient**	**95% CI**	**P**
Cp	0.08158	0.04391 to 0.1192	<0.0001	0.04496	0.01264 to 0.07728	0.0066
Fn	0.0959	0.07678 to 0.1150	<0.0001	0.0723	0.05297 to 0.09163	<0.0001
m3	0.07913	0.05452 to 0.1038	<0.0001	0.03554	0.01284 to 0.05824	0.0022
Ch	0.1478	0.09391 to 0.2018	<0.0001	0.09195	0.04454 to 0.1394	0.0002
Bc	-0.03751	-0.07358 to -0.001446	0.0415	-0.03072	-0.06061 to -0.0008275	0.044
**Age**	0.04378	0.02975 to 0.05781	<0.0001	0.02064	0.008007 to 0.03327	0.0014
**Gender**	-0.204	-0.4616 to 0.05357	0.1202	-0.161	-0.3717 to 0.04983	0.134
**BMI**	0.1465	-0.02316 to 0.3163	0.0881			

**Cp*, *Cloacibacillus porcorum*; *Fn*, *Fusobacterium nucleatum*; *m3*, marker m3; *Ch*, *Clostridium hathewayi*; *Bc*, *Bacteroides clarus*; BMI, body mass index.

Table S5. Logistic regression analysis for diagnosis of CRC and adenoma

Variable*	Adenoma vs N				CRC vs N			
	**Coefficient**	**Std. Error**	**Wald**	**P**	**Coefficient**	**Std. Error**	**Wald**	**P**
Cp	0.5344	0.20074	7.0868	0.0078	0.44209	0.20706	4.5585	0.0328
Fn	0.082991	0.038422	4.6655	0.0308	0.22352	0.043443	26.4737	<0.0001
m3	0.18911	0.048067	15.4783	0.0001	0.16938	0.059437	8.1214	0.0044
Ch	0.11306	0.24133	0.2195	0.6394	0.65969	0.2548	6.7032	0.0096
Bc	-0.039305	0.04108	0.9154	0.3387	-0.083451	0.067324	1.5365	0.2151
**Age**	0.054743	0.028414	3.7118	0.054	0.080075	0.029468	7.3842	0.0066
**Gender**	-0.52146	0.33207	2.466	0.1163	-0.54817	0.47827	1.3137	0.2517

**Cp*, *Cloacibacillus porcorum*; *Fn*, *Fusobacterium nucleatum*; *m3*, marker m3; *Ch*, *Clostridium hathewayi*; *Bc*, *Bacteroides clarus*; N, normal control.

Table S6. Combination of markers for diagnosis of CRC and adenoma.

**Model***	**Markers****	**CRC&Adenoma vs N**				**CRC vs N**				**Adenoma vs N**			
		**AUC**	**SE**	**95% CI**	***P***	**AUC**	**SE**	**95% CI**	***P***	**AUC**	**SE**	**95% CI**	***P***
**LR2**	**Fn, m3,** ~~Ch, Cp, Bc~~	0.791	0.027	0.742 to 0.834	<0.0001	0.887	0.024	0.832 to 0.929	<0.0001	0.720	0.035	0.656 to 0.778	<0.0001
**LR3**	**Fn, m3,** ~~Ch,~~ **Cp**~~, Bc~~	0.824	0.025	0.778 to 0.864	<0.0001	0.897	0.023	0.844 to 0.937	<0.0001	0.770	0.032	0.709 to 0.824	<0.0001
**LR3'**	**Fn, m3, Ch**~~, Cp, Bc~~	0.800	0.026	0.753 to 0.842	<0.0001	0.911	0.021	0.860 to 0.948	<0.0001	0.719	0.035	0.655 to 0.777	<0.0001
**LR4**	**Fn, m3,** ~~Ch,~~ **Cp, Bc**	0.826	0.025	0.780 to 0.866	<0.0001	0.901	0.023	0.848 to 0.940	<0.0001	0.771	0.032	0.710 to 0.825	<0.0001
**4Bac**	**Fn, m3, Ch,** ~~Cp,~~ **Bc**	0.797	0.026	0.749 to 0.840	<0.0001	0.920	0.020	0.870 to 0.955	<0.0001	0.707	0.036	0.643 to 0.766	<0.0001
**LR5**	**Fn, m3, Ch, Cp, Bc**	0.833	0.024	0.788 to 0.872	<0.0001	0.923	0.020	0.875 to 0.957	<0.0001	0.767	0.032	0.706 to 0.821	<0.0001
**LR2&FIT**	**Fn, m3,** ~~Ch, Cp, Bc~~**, FIT**	0.844	0.022	0.800 to 0.882	<0.0001	0.970	0.012	0.934 to 0.990	<0.0001	0.752	0.033	0.689 to 0.807	<0.0001
**LR3&FIT**	**Fn, m3,** ~~Ch,~~ **Cp**~~, Bc~~**, FIT**	0.871	0.020	0.829 to 0.905	<0.0001	0.970	0.012	0.933 to 0.989	<0.0001	0.798	0.030	0.739 to 0.849	<0.0001
**LR3'&FIT**	**Fn, m3, Ch**~~, Cp, Bc~~**, FIT**	0.847	0.022	0.804 to 0.885	<0.0001	0.976	0.010	0.942 to 0.993	<0.0001	0.753	0.033	0.691 to 0.808	<0.0001
**LR4&FIT**	**Fn, m3,** ~~Ch,~~ **Cp, Bc, FIT**	0.874	0.020	0.832 to 0.908	<0.0001	0.970	0.012	0.934 to 0.990	<0.0001	0.802	0.030	0.744 to 0.853	<0.0001
**4Bac&FIT**	**Fn, m3, Ch,** ~~Cp,~~ **Bc, FIT**	0.844	0.022	0.800 to 0.882	<0.0001	0.977	0.010	0.944 to 0.994	<0.0001	0.747	0.034	0.684 to 0.803	<0.0001
**5Bac&FIT**	**Fn, m3, Ch, Cp, Bc, FIT**	0.875	0.019	0.834 to 0.908	<0.0001	0.975	0.011	0.941 to 0.993	<0.0001	0.800	0.030	0.742 to 0.851	<0.0001

*LR, logistic regression. ***Fn*, *Fusobacterium nucleatum*; *m3*, marker m3; *Ch*, *Clostridium hathewayi*; *Cp*, *Cloacibacillus porcorum*; *Bc*, *Bacteroides clarus*; FIT, fecal immunochemical test.

Table S7. Nucleotide sequences of primers and probed used in this study.

**Primers***	**sequence (5'-->3')**
Bc-F	TCCATCCGCAAGCCTTTACT
Bc-R	GCTTCCGGTGCCATTGACTA
m3-F	AATGGGAATGGAGCGGATTC
m3-R	CCTGCACCAGCTTATCGTCAA
Ch-F	GGGCTGCGGAAGCAACTTA
Ch-R	GATGACCTCGCCCTGATCAT
Fn-F	TTCAATAAAAGTGGCAGGTCAAG
Fn-R	TAACAACACATGCAGGTCAATGG
Cp-F	GGCTCCGCAAGCTTTACAAT
Cp-R	CATGCTCGCGAAGTCTGTCA
I. butyriciproducens-F	ACCGCGTTTGGTATTTTCGT
I. butyriciproducens-R	CTGAAAGGCATCGCCGATAT
C-F	CGTCAGCTCGTGYCGTGAG
C-R	CGTCRTCCCCRCCTTCC
**Probes**	**sequence (5'-->3')**
Bc	TTCATCATCACAGCCGACAACGCA
m3	AAGCCTGCGGAACCACAGTTACCAGC
Ch	ACCACCACACAGGACGGAAAGATTCTCC
Fn	ACTCGAACCCCCAACCCTCGGTTT
Cp	TCGAAGGGCGCAACAACAACCAT
I. butyriciproducens	TTCCGGCATAAAAGCGACGACAAAGC
C	TTAAGTCCCRYAACGAGCGCAACCC

**Fn*, *Fusobacterium nucleatum*; *Ch*, *Clostridium hathewayi*; *m3*, marker m3; *Cp*, *Cloacibacillus porcorum*; C, universal 16 rDNA control.

Table S8. Examples of fecal bacterial markers combination and their corresponding calculation methods for non-invasive diagnosis of CRC and adenoma.

Model*	Markers**	Algorithms
**4Bac**	**Fn, m3, Ch, ~~Cp,~~ Bc**	$I_1 + \beta_1 * Fn + \beta_2 * m3 + \beta_3 * Bc + \beta_4 * Ch$
**LR2**	**Fn, m3,** ~~Ch, Cp, Bc~~	$I_2 + \beta_5 * Fn + \beta_6 * m3$
**LR3**	**Fn, m3,** ~~Ch,~~ **Cp**~~, Bc~~	$I_3 + \beta_7 * Fn + \beta_8 * m3 + \beta_9 * Cp$
**LR3’**	**Fn, m3, Ch**~~, Cp, Bc~~	$I_4 + \beta_{10} * Fn + \beta_{11} * m3 + \beta_{12} * Ch$
**LR4**	**Fn, m3,** ~~Ch,~~ **Cp, Bc**	$I_5 + \beta_{13} * Fn + \beta_{14} * m3 + \beta_{15} * Cp + \beta_{16} * Bc$
**LR5**	**Fn, m3, Ch, Cp, Bc**	$I_6 + \beta_{17} * Fn + \beta_{18} * m3 + \beta_{19} * Ch + \beta_{20} * Cp + \beta_{21} * Bc$
**LR2&FIT**	**Fn, m3,** ~~Ch, Cp, Bc~~**, FIT**	$I_7 + \beta_{22} * Fn + \beta_{23} * m3 + \beta_{24} * \mathrm{FIT}$
**LR3&FIT**	**Fn, m3,** ~~Ch,~~ **Cp**~~, Bc~~**, FIT**	$I_8 + \beta_{25} * Fn + \beta_{26} * m3 + \beta_{27} * Cp + \beta_{28} * \mathrm{FIT}$
**LR3’&FIT**	**Fn, m3, Ch**~~, Cp, Bc~~**, FIT**	$I_9 + \beta_{29} * Fn + \beta_{30} * m3 + \beta_{31} * Ch + \beta_{32} * \mathrm{FIT}$
**LR4&FIT**	**Fn, m3,** ~~Ch,~~ **Cp, Bc, FIT**	$I_{10} + \beta_{33} * Fn + \beta_{34} * m3 + \beta_{35} * Cp + \beta_{36} * Bc + \beta_{37} * \mathrm{FIT}$
**4Bac&FIT**	**Fn, m3, Ch,** ~~Cp,~~ **Bc, FIT**	$I_{11} + \beta_{38} * Fn + \beta_{39} * m3 + \beta_{40} * Ch + \beta_{41} * Bc + \beta_{42} * \mathrm{FIT}$
**5Bac&FIT**	**Fn, m3, Ch, Cp, Bc, FIT**	$I_{12} + \beta_{43} * Fn + \beta_{44} * m3 + \beta_{45} * Ch + \beta_{46} * Cp + \beta_{47} * Bc + \beta_{48} * \mathrm{FIT}$

*LR, logistic regression. ***Fn*, *Fusobacterium nucleatum*; *m3*, marker m3; *Ch*, *Clostridium hathewayi*; *Cp*, *Cloacibacillus porcorum*; *Bc*, *Bacteroides clarus*; FIT, fecal immunochemical test.
