# Supplementary figures and images for: Fecal Cloacibacillus porcorum Improves Non-Invasive Diagnosis of Colorectal Adenoma in the Hong Kong Population
Source: Int J Mol Sci. 2026 May 15;27(10):4457. doi: 10.3390/ijms27104457 (PMC13207295; doi:10.3390/ijms27104457)

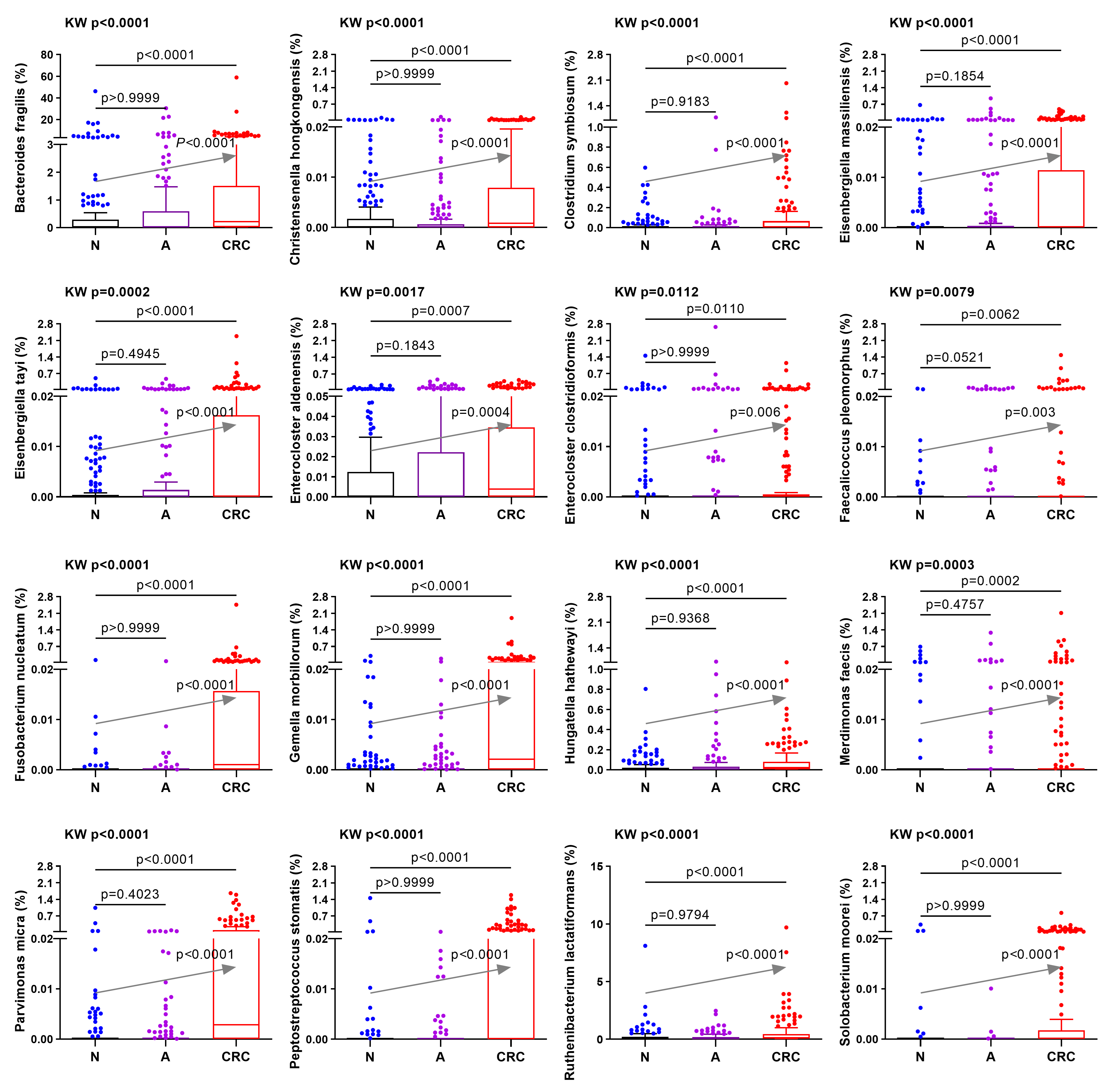

Supplement: Supplementary file 1 [file ijms-27-04457-s001.zip › Figure S1.tif]
